# Temporal dynamics of fibrolytic and methanogenic rumen microorganisms during *in situ* incubation of switchgrass determined by 16S rRNA gene profiling

**DOI:** 10.3389/fmicb.2014.00307

**Published:** 2014-07-22

**Authors:** Hailan Piao, Medora Lachman, Stephanie Malfatti, Alexander Sczyrba, Bernhard Knierim, Manfred Auer, Susannah G. Tringe, Roderick I. Mackie, Carl J. Yeoman, Matthias Hess

**Affiliations:** ^1^Systems Microbiology and Biotechnology Group, School of Molecular Biosciences, Washington State UniversityRichland, WA, USA; ^2^Department of Animal and Range Sciences, Montana State UniversityBozeman, MT, USA; ^3^Lawrence Livermore National Laboratory, Biosciences and Biotechnology DivisionLivermore, CA, USA; ^4^Faculty of Technology and Center for Biotechnology, Bielefeld UniversityBielefeld, Germany; ^5^Lawrence Berkeley National Laboratory, Life Sciences DivisionBerkeley, CA, USA; ^6^Prokaryote Super Program, DOE Joint Genome InstituteWalnut Creek, CA, USA; ^7^Department of Animal Sciences and Institute for Genomic Biology, University of Illinois, Urbana-ChampaignIL, USA; ^8^Energy and Efficiency Division, Chemical and Biological Process Development Group, Pacific Northwest National LaboratoryRichland, WA, USA

**Keywords:** rumen microbiology, microbe-microbe interactions, cellulolytic bacteria, methanogenic archaea, interspecies H_2_ transfer

## Abstract

The rumen microbial ecosystem is known for its biomass-degrading and methane-producing phenotype. Fermentation of recalcitrant plant material, comprised of a multitude of interwoven fibers, necessitates the synergistic activity of diverse microbial taxonomic groups that inhabit the anaerobic rumen ecosystem. Although interspecies hydrogen (H_2_) transfer, a process during which bacterially generated H_2_ is transferred to methanogenic *Archaea*, has obtained significant attention over the last decades, the temporal variation of the different taxa involved in *in situ* biomass-degradation, H_2_ transfer and the methanogenesis process remains to be established. Here we investigated the temporal succession of microbial taxa and its effect on fiber composition during rumen incubation using 16S rRNA amplicon sequencing. Switchgrass filled nylon bags were placed in the rumen of a cannulated cow and collected at nine time points for DNA extraction and 16S pyrotag profiling. The microbial community colonizing the air-dried and non-incubated (0 h) switchgrass was dominated by members of the *Bacilli* (recruiting 63% of the pyrotag reads). During *in situ* incubation of the switchgrass, two major shifts in the community composition were observed: *Bacilli* were replaced within 30 min by members belonging to the *Bacteroidia* and *Clostridia*, which recruited 34 and 25% of the 16S rRNA reads generated, respectively. A second significant shift was observed after 16 h of rumen incubation, when members of the *Spirochaetes* and *Fibrobacteria* classes became more abundant in the fiber-adherent community. During the first 30 min of rumen incubation ~13% of the switchgrass dry matter was degraded, whereas little biomass degradation appeared to have occurred between 30 min and 4 h after the switchgrass was placed in the rumen. Interestingly, methanogenic members of the *Euryarchaeota* (i.e., *Methanobacteria*) increased up to 3-fold during this period of reduced biomass-degradation, with peak abundance just before rates of dry matter degradation increased again. We hypothesize that during this period microbial-mediated fibrolysis was temporarily inhibited until H_2_ was metabolized into CH_4_ by methanogens. Collectively, our results demonstrate the importance of inter-species interactions for the biomass-degrading and methane-producing phenotype of the rumen microbiome—both microbially facilitated processes with global significance.

## Introduction

The microbial community (microbiota) inhabiting the cow rumen has been described as “the most elegant and highly evolved cellulose-digesting system in nature” (Weimer et al., 2009). Cellulose, the most abundant natural polymer on earth (Klemm et al., 2005), and a major component of plant biomass (Heredia et al., 1995) is degraded within the rumen by various bacteria (Weimer, 1996), fungi (Bauchop and Mountfort, 1981; Theodorou et al., 1996), and protozoa (Coleman, 1992). The efficacy of cellulose-degradation, and ultimately fiber-degradation, is mediated by important microbial enzymatic and metabolic interactions and it has been shown that major cellulolytic bacteria, such as *Fibrobacter succinogenes, Ruminococcus flavefaciens*, and *Ruminococcus albus* (Weimer, 1996), exhibit suboptimal cellulolytic activity if they are not able to synergistically engage with non-cellulolytic microorganisms (Kudo et al., 1987; Fondevila and Dehority, 1996). In particular methanogenic archaea have been described to boost the carbohydrate-degrading activity of cellulolytic rumen bacteria (Joblin et al., 1989). The role of rumen methanogens is unique in that these organisms do not degrade or ferment any portion of plant biomass, but instead obtain their energy from byproducts, principally H_2_ and CO_2_, of fibrolytic organisms (Janssen and Kirs, 2008). By using hydrogen to reduce CO_2_, methanogens remove otherwise inhibitory levels of H_2_ (Janssen and Kirs, 2008), increase ATP yields by redirecting reducing equivalents toward acetate (Latham and Wolin, 1977) and thereby promote growth (Rychlik and May, 2000) and the ability to produce higher concentrations of fibrolytic enzymes. Physical co-aggregation between fibrolytic and methanogenic populations are commonly observed (i.e., Leahy et al., 2010) and this is thought to be important for both the efficient transfer of H_2_ and maintaining the low H_2_ partial pressures necessary to sustain active growth of the fermentative bacteria (Stams, 1994; Ishii et al., 2006).

To this end, the temporal dynamics of fibrolytic bacteria and methanogenic archaea remain to be systematically explored during *in situ* colonization and degradation of plant biomass within the rumen. Previous studies have shown temporal changes in bacterial (Edwards et al., 2007; Huws et al., 2013) and fungal (Edwards et al., 2008) populations during ruminal incubation of fresh perennial rye grass (*Lolium perenne*). Specifically, these studies revealed a rapid colonization of the plant material within 5 min of rumen-incubation by both bacterial (Edwards et al., 2007) and fungal populations, followed by a compositional shift in bacterial populations between 2 and 4 h following incubation (Huws et al., 2013). Compositional dynamics may vary among forage types based on observed differences in fiber disappearance and fibrolytic enzyme activities (Bowman and Firkins, 1993) and the observed temporal changes in microbiota may be affected by residual plant metabolism of fresh forages (Huws et al., 2013). Here we provide evidence that similar temporal changes occur during the *in situ* incubation of dried switchgrass and correspond to changes in methanogen abundance. Furthermore, these changes in microbiota also correspond to notable differences in the rate of forage degradation.

## Materials and methods

### Sample collection

To enrich for fiber-adherent rumen microorganisms, air-dried switchgrass (*Panicum virgatum*) was ground into 2 mm pieces using a Wiley mill and weighed into individual *in situ* nylon bags (50 μm pores; Ankom Technology, Macedon, NY, USA). A total of 18 nylon bags, each containing 5 g of air-dried switchgrass, were placed in the rumen of one cannulated Friesian cow, fed on a mixed diet containing 60% fiber (Hess et al., 2011). Nylon bags were retrieved from the cow's rumen at 0.5, 1, 2, 4, 6, 16, 24, 48, and 72 h and washed immediately with 2 × 50 ml PBS buffer (pH7) to remove rumen fluid containing loosely adherent microbes, placed on dry-ice and transported immediately to the laboratory where DNA extraction and fiber degradation analysis were performed. Non-incubated nylon bags filled with ground switchgrass were used as control (0 h). To account for biological variation during the fiber colonization process, nylon bags were retrieved from the cow's rumen in duplicates. All animal procedures were carried out under an approved protocol with the University of Illinois Institutional Animal Care and Use of Animals Committee (IUCAC #06081).

### Fiber degradation analysis

Relative biomass (switchgrass) degradation during rumen-incubation was determined by dry matter, organic matter, neutral detergent fiber (NDF), acid detergent fiber (ADF), and acid detergent lignin (ADL) analysis. NDF, ADF, and ADL were determined using the procedures of Goering and Van Soest (1970). Cellulose content was estimated as ADF–ADL and hemicellulose as NDF–ADF.

### DNA extraction and 16S rRNA gene amplification

Total microbial genomic DNA was extracted from 100 mg of the non-incubated control sample and from each rumen-incubated biomass sample using a FastDNA SPIN Kit for Soil (MP Biomedical, Solon, OH) according to the manufacturer's instructions. Extracted DNA was quantified with a spectrophotometer (Nanodrop ND1000; Thermo Scientific, USA). The hypervariable V6 to V8 region of the 16S ribosomal RNA (rRNA) gene was amplified from the environmental DNA using the primer set 926F/1392R (926F: 5′- cct atc ccc tgt gtg cct tgg cag tct cag AAA CTY AAA KGA ATT GRC GG- 3′ and 1392R: 5′ - cca tct cat ccc tgc gtg tct ccg act cag xxxxx ACG GGC GGT GTG TRC – 3′) described by Engelbrektson et al. (2010). Primer sequences were modified by the addition of 454 A or B adapter sequences (lower case). In addition, the reverse primer included a 5 bp barcode, indicated by xxxxx in the reverse primer sequence above, for multiplexing of samples during sequencing. The barcode sequence for each sample is listed in Table 1. The generated raw reads were deposited in NCBI's Short Read Archive under the accession number SRP042121.

**Table 1:** **Barcode sequences**.

**Sample ID**	0h_A	0h_B	0.5h_A	0.5h_B	1h_A	1h_B	2h_A	2h_B	4h_A	4h_B
**Barcode sequence**	TGTAG	ATATG	CTACT	CATGC	CTGCG	CGATG	CGTAC	CACAG	TACTG	CGATG
**Sample ID**	6h_A	6h_B	16h_A	16h_B	24h_A	24h_B	48h_A	48h_B	72h_A	72h_B
**Barcode sequence**	TAGAG	TCTCG	TCATC	AGCAC	TCTAT	CACAG	TGCTG	CATGC	ATGCT	CACAG

Twenty-microliter PCR reactions were performed in duplicate and pooled to minimize PCR bias. PCR reaction was performed using 0.4 μl Advantage GC 2 Polymerase Mix (Advantage-2 GC PCR Kit, Clontech), 4 μl 5x GC PCR buffer, 2 μl 5 M GC Melt Solution, 0.4 μl 10 mM dNTP mix (MBI Fermentas), 1.0 μl of each 25 nM primer, and 10 ng sample DNA. The thermal cycler protocol was 95°C for 3 min, 25 cycles of 95°C for 30 s, 50°C for 45 s, and 68°C for 90 s, and a final 10 min extension at 68°C. PCR amplicons were purified using Solid Phase Reversible Immobilization (SPRI) beads and quantified using a Qubit flurometer (Invitrogen). Samples were diluted to 10 ng/μl and mixed in equal concentrations. Emulsion PCR and sequencing of the PCR amplicons were performed following the Roche 454 GS FLX Titanium technology instructions provided by the manufacturer.

### Pyrotag sequence analysis

Pyrosequencing data from the 20 samples were demultiplexed and processed using QIIME version 1.7.0 (Caporaso et al., 2010) according to the standard operating procedure described at http://qiime.org/tutorials/tutorial.html. Primers and barcodes were removed before the raw reads were quality filtered. Sequences were removed if they had long homopolymeric regions (>6 nt), were smaller than 200 nt, had quality scores lower than 25, or if they were identified as being chimeric. This resulted in a total of 198,037 high quality 16S rRNA gene sequences. Sequences generated from each of the biological duplicates were combined prior to sequence analysis to account for biological variation.

### OTU-based sequence analysis

Sequences were clustered into operational taxonomic units (OTUs) at a 97% sequence identity cut-off using UCLUST (Edgar, 2010). The most abundant sequence of each OTU was picked as representative sequence. Singleton and doubleton abundance, Shannon, Simpson, Simpson reciprocal, and Chao1 estimators were calculated using the QIIME software. Representative sequences were aligned using the PyNAST algorithm and the alignment was filtered to remove common gaps. Following the quality filtering and grouping steps, 7,168 unique sequences (representing 198,037 total sequences) were used for OTU analyses. QIIME scripts were used to calculate α-diversity metrics for OTU representative sequences and to generate a Principal Coordinate Analysis plot according to the standard operating procedure described at http://qiime.org/tutorials/tutorial.html.

### Taxonomic classification of unique representative sequences

Taxonomic classification of the final set of representative sequences was performed using QIIME. Each sequence was normalized to contain six taxonomic levels, ranging from the domain to the genus level.

### Scanning electron microscopy

For scanning electron microscopy (SEM) pieces of switchgrass taken from the cow rumen as described above were immediately fixed with glutaraldehyde. The samples were washed several times with phosphate buffered saline, treated with 1% OsO_4_ for 1 h on ice, prior to dehydration and critical point drying using a Tousimis Autosamdri-815 critical point dryer. The dried samples were then mounted on precut brass sample stubs using double sided carbon tape and sputter coated with approximately 30 Angstrom of Au/Pd. SEM imaging was performed on a Hitachi S-5000 microscope at 10 kV accelerating voltage.

## Results

### Temporal changes in α-diversity of fiber-adherent rumen microbiome

**Figure 1:**
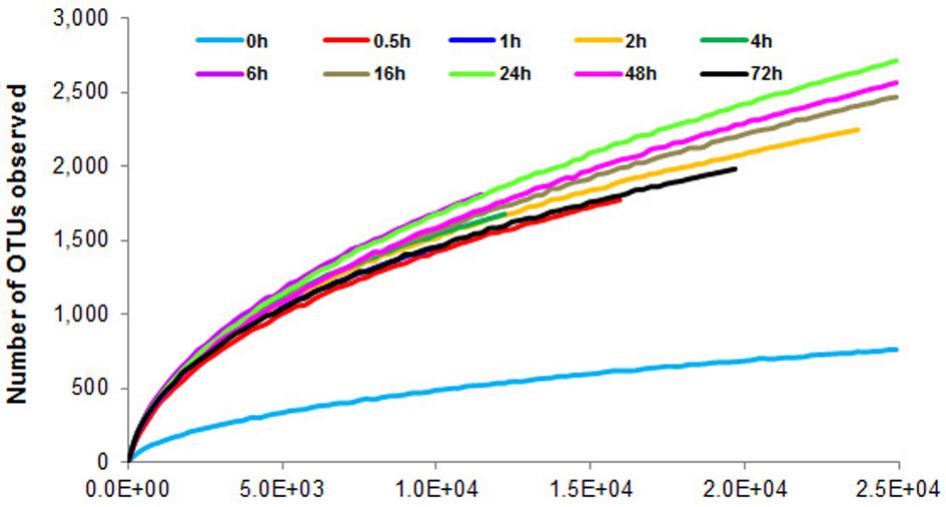
**Rarefaction curves calculated from 16S rRNA pyrotag data of switchgrass-adherent microbiomes after rumen-incubation**. 0 h indicates the non-incubated switchgrass sample (control).

To determine α-diversity within the fiber-adherent community the quality-filtered pyrotag reads of each sample were clustered into OTUs at a sequence identify level of 97%. This resulted in a total of 7,168 distinct OTUs, with a range of 788–2,758 OTUs observed for each community. Table 2 summarizes the number of raw reads, quality filtered reads, and OTUs that were obtained for each time point. Of the observed 7,168 OTUs, 7,005 (97.7%) were determined to be unambiguously of bacterial origin, while 0.2% (17 OTUs) were assigned to be archaeal (Table 3). Table 4 summarizes the number of OTUs unique to each time point sampled. A complete list of all OTUs observed during this study and an OTU count for each sample is provided in Supplementary Table 1. Analysis of the successional appearance of OTUs suggests an initial increase in community richness within the first 30 min of rumen incubation (Tables 2, 3) and a second increase in richness of the bacterial community associated with the switchgrass samples subjected to rumen-incubation for 16, 24, and 48 h (Table 3). This finding is supported by the calculated rarefaction curves (Figure 1) and Chao 1 indices (Table 5). Likewise the Shannon and Simpson Reciprocal indices increased noticeably for all rumen-incubated samples (Table 5). An average Good's coverage estimator of 94.9% was calculated for all samples, with 92% (6 h) being the lowest and 98.6% (non-incubated switchgrass) being the highest (Table 5).

**Table 2:** **Summary of pyrosequencing reads generated and OTUs observed for each time point sampled during this study**.

**Incubation time [h]**	**0**	**0.5**	**1**	**2**	**4**	**6**	**16**	**24**	**48**	**72**	**Total count**
Raw reads	30,150	22,165	14,104	33,037	16,966	15,441	34,313	34,459	33,850	28,216	262,701
Quality filtered reads	26,888	16,119	10,270	23,892	12,234	11,690	26,059	25,771	25,373	19,741	198,037
OTUs observed	788	1,782	1,470	2,257	1,679	1,827	2,524	2,758	2,589	1,985	7,168

**Table 3:** **Phylogenetic assignment of OTUs at domain level**.

**Incubation time [h]**	**0**	**0.5**	**1**	**2**	**4**	**6**	**16**	**24**	**48**	**72**	**Total OTU count**
Archaea	2	7	8	8	8	6	7	6	5	11	17
Bacteria	703	1,740	1,448	2,221	1,636	1,809	2,505	2,741	2,562	1,940	7,005
Unclassified	83	35	13	27	35	12	12	11	22	34	146
Number of OTUs observed	788	1,782	1,469	2,256	1,679	1,827	2,524	2,758	2,589	1,985	7,168

**Table 4:** **Distinct OTUs observed**.

**Incubation time [h]**	**0 h**	**0.5 h**	**1 h**	**2 h**	**4 h**	**6 h**	**16 h**	**24 h**	**48 h**	**72 h**
Number of distinct OTUs observed	260	289	180	358	224	308	563	727	689	296

**Table 5:** **Estimated OTU richness and diversity metrics and estimated sample coverage of rarefied bacterial and archaeal 16S rRNA sequences**.

**Incubation time [h]**	**0**	**0.5**	**1**	**2**	**4**	**6**	**16**	**24**	**48**	**72**
Sequences sampled	24,910	15,946	10,219	23,665	12,211	11,464	24,910	24,910	24,910	19,681
Observed species	760.5	1,771.5	1,466.2	2,248.1	1,678.0	1,809.8	2,468.3	2,714.8	2,565.5	1,982.6
Goods coverage (%)	98.6	95.0	93.8	95.9	94.1	92.0	95.2	94.4	94.8	95.6
Singles	352.7	800.5	635.2	978.2	718.6	922.4	1,202.7	1,384.3	1,303.0	861.2
Doubles	124.9	285.7	212.2	305.9	261.3	253.3	332.0	398.6	376.8	293.6
Chao1	1,253.6	2,888.0	2,411.1	3,805.8	2,661.0	3,481.2	4,639.3	5,111.3	4,811.2	3,239.9
Shannon	4.0	7.9	8.9	9.0	8.9	9.0	8.9	8.9	8.8	8.9
Simpson	0.7	1.0	1.0	1.0	1.0	1.0	1.0	1.0	1.0	1.0
Simpson reciprocal	3.8	23.6	204.2	189.0	160.2	202.5	135.5	126.6	122.9	183.7

### Temporal changes in β-diversity of fiber-adherent rumen microbiome

**Figure 2:**
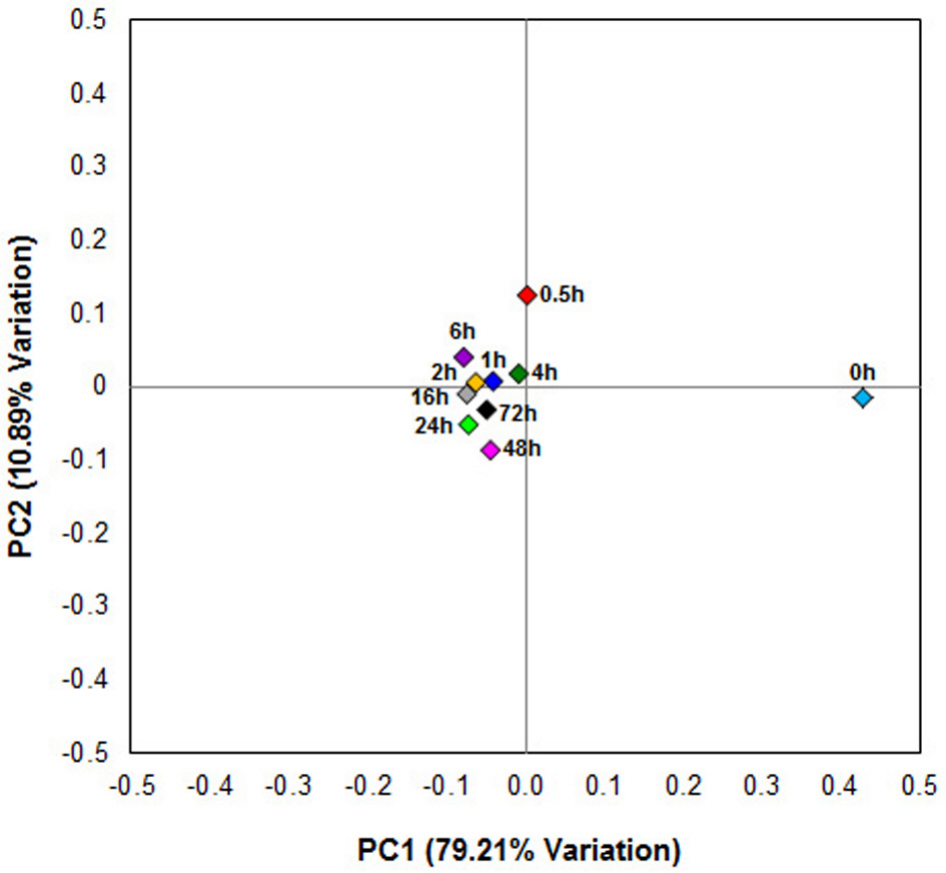
**Principal coordinate analysis of switchgrass-adherent microbiomes**. Each point corresponds to one of nine rumen-incubated samples (0.5, 1, 2, 4, 6, 16, 24, 48, and 72 h) and one control sample (0 h). The percentage of variation explained by the plotted principal coordinates is indicated on the axes.

Principal coordinates analysis comparing the temporal dynamics of switchgrass-associated microbiota indicated that the community associated with the air-dried switchgrass sample (0 h) was distinct from the microbiomes of all rumen-incubated samples (Figure 2). After 30 min of rumen incubation the switchgrass-associated microbiota exhibited clearly noticeable changes: for example, members of the *Archaea*, which were essentially absent in the non-incubated microbiome, were detected in all rumen-incubated samples. In total, 17 distinct archaeal OTUs were detected (Table 3), with 14 (82%) of them categorized as *Methanobacteriaceae*, a family belonging to the methanogenic *Euryarchaeota* (Supplementary Table 1). Of the reads derived from the *Archaea*, the majority (≥76%) was recruited for each sample (median 88%) by two OTUs both categorized as *Methanobrevibacter* following rumen incubation. Abundance of members belonging to the genus *Methylobacterium* was notably high in the samples retrieved after 30 min of rumen incubation. The bacterial phylum *Bacteroidetes*, represented primarily by members of the *Sphingobacteria* in the 0 h sample, increased up to five fold throughout rumen incubation, mainly through large increases in *Bacteroidia* populations and in particular of increases in members belonging to the genus *Prevotella* (Tables 6–8). This overall increase in *Bacteroidetes* was accompanied by increased abundance of *Cyanobacteria, Fibrobacteres, Spirochaetes*, and *Tenericutes* and decreases in *Actinobacteria* (i.e., *Kineosporiaceae* and *Microbacteriaceae*), *Firmicutes, and Proteobacteria* abundance (Table 8).

**Table 6:** **Community composition of switchgrass-adherent microbiome at the class level based on 16S rRNA pyrotag data**.

	**Percentage of sequences**									
	**0 h**	**0.5 h**	**1 h**	**2 h**	**4 h**	**6 h**	**16 h**	**24 h**	**48 h**	**72 h**
**BACTERIA**										
Actinobacteria;c__Actinobacteria	3.77	0.60	0.39	0.48	0.70	0.28	0.20	0.23	0.35	0.43
Bacteroidetes;c__Bacteroidia	2.31	33.87	31.93	36.65	27.90	36.77	34.94	35.13	32.22	34.30
Bacteroidetes;c__Flavobacteriia	0.63	0.03	0.01	0.00	0.00	0.01	0.00	0.00	0.00	0.00
Bacteroidetes;c__Sphingobacteriia	4.34	0.13	0.03	0.05	0.04	0.03	0.02	0.00	0.00	0.03
Chloroflexi;c__Anaerolineae	0.01	0.13	0.35	0.33	0.23	0.15	0.26	0.29	0.38	0.35
Cyanobacteria;c__4C0d-2	0.01	0.90	1.48	0.80	1.30	0.82	0.97	0.65	0.33	0.95
Elusimicrobia;c__Elusimicrobia	0.01	0.23	0.21	0.08	0.13	0.09	0.07	0.07	0.04	0.06
Elusimicrobia;c__Endomicrobia	0.02	0.18	0.19	0.15	0.18	0.16	0.22	0.19	0.23	0.20
Fibrobacteres;c__Fibrobacteria	0.11	0.35	1.35	2.14	0.63	2.30	6.87	7.54	6.23	2.11
Firmicutes;c__Bacilli	63.26	0.11	0.12	0.05	0.03	0.22	0.02	0.06	0.07	0.04
Firmicutes;c__Clostridia	1.91	24.65	41.64	42.54	43.10	38.86	37.29	38.95	42.94	42.96
Firmicutes;c__Erysipelotrichi	0.02	0.09	0.10	0.26	0.23	0.20	0.18	0.22	0.32	0.21
Firmicutes;c__RF3	0.03	0.32	0.36	0.55	0.41	0.28	0.43	0.45	0.48	0.53
Lentisphaerae;c__[Lentisphaeria]	0.02	0.34	0.79	0.55	0.58	0.51	0.60	0.39	0.58	0.71
Planctomycetes;c__Planctomycetia	0.01	0.25	0.53	0.50	0.49	0.26	0.28	0.38	0.62	0.60
Proteobacteria;c__Alphaproteobacteria	12.93	22.76	1.31	0.90	1.79	0.88	0.63	0.40	0.44	0.81
Proteobacteria;c__Betaproteobacteria	5.51	0.22	0.16	0.11	0.47	0.14	0.01	0.02	0.02	0.06
Proteobacteria;c__Deltaproteobacteria	0.03	0.09	0.32	0.26	0.23	0.31	0.38	0.48	0.42	0.30
Proteobacteria;c__Gammaproteobacteria	4.28	2.28	4.11	2.09	3.00	4.57	3.23	1.78	1.49	1.79
Spirochaetes;c__Spirochaetes	0.23	1.78	2.03	1.62	1.21	2.68	4.40	4.58	3.70	2.18
Synergistetes;c__Synergistia	0.00	0.03	0.18	0.06	0.06	0.13	0.12	0.09	0.15	0.10
Tenericutes;c__Mollicutes	0.21	7.46	6.18	5.10	10.34	5.78	4.60	3.47	3.17	4.61
TM7;c__TM7-3	0.02	0.06	0.17	0.03	0.26	0.05	0.04	0.00	0.03	0.03
Verrucomicrobia;c__Verruco-5	0.01	0.32	0.49	0.47	0.54	0.35	0.31	0.25	0.25	0.60
Bacteria classes <0.1%	0.11	0.11	0.18	0.16	0.14	0.09	0.13	0.09	0.23	0.20
Unclassified bacteria classes	0.20	2.42	4.73	3.56	5.06	3.67	3.55	4.09	5.00	5.11
**ARCHAEA**										
Euryarchaeota;c__Methanobacteria	0.01	0.28	0.61	0.47	0.91	0.34	0.21	0.20	0.30	0.69
Archaea classes <0.1%	0.00	0.01	0.06	0.03	0.05	0.09	0.02	0.02	0.01	0.05

**Table 7:** **Community composition of switchgrass-adherent microbiome at the genus level based on 16S rRNA pyrotag data**.

	**Percentage of sequences**									
	**0 h**	**0.5 h**	**1 h**	**2 h**	**4 h**	**6 h**	**16 h**	**24 h**	**48 h**	**72 h**
**BACTERIA**										
Actinobacteria;c__Actinobacteria;o__Actinomycetales; f__Microbacteriaceae;g__Microbacterium	0.21	0.04	0.01	0.00	0.04	0.00	0.00	0.00	0.00	0.01
Actinobacteria;c__Actinobacteria;o__Actinomycetales; f__Propionibacteriaceae;g__Propionibacterium	0.01	0.11	0.00	0.00	0.00	0.00	0.03	0.00	0.00	0.00
Actinobacteria;c__Actinobacteria;o__Actinomycetales; f__Pseudonocardiaceae;g__Saccharopolyspora	0.02	0.03	0.14	0.25	0.16	0.08	0.09	0.16	0.20	0.21
Bacteroidetes;c__Bacteroidia;o__Bacteroidales; f__Bacteroidaceae;g__BF311	0.06	0.63	1.06	1.37	0.80	0.92	1.54	1.67	1.91	1.46
Bacteroidetes;c__Bacteroidia;o__Bacteroidales; f__Porphyromonadaceae;g__Paludibacter	0.04	2.21	1.03	1.36	1.53	1.08	0.86	0.53	0.40	0.88
Bacteroidetes;c__Bacteroidia;o__Bacteroidales; f__Prevotellaceae;g__Prevotella	1.21	18.25	14.09	14.60	12.79	19.68	15.59	13.12	9.40	12.30
Bacteroidetes;c__Bacteroidia;o__Bacteroidales; f__[Paraprevotellaceae];g__CF231	0.03	0.37	0.53	0.49	0.42	0.68	0.70	0.67	0.39	0.43
Bacteroidetes;c__Bacteroidia;o__Bacteroidales; f__[Paraprevotellaceae];g__YRC22	0.10	1.19	1.23	1.14	0.93	1.49	1.36	1.20	0.84	1.15
Bacteroidetes;c__Flavobacteriia;o__Flavobacteriales; f__Flavobacteriaceae;g__Chryseobacterium	0.21	0.01	0.00	0.00	0.00	0.01	0.00	0.00	0.00	0.00
Bacteroidetes;c__Sphingobacteriia;o__Sphingobacteriales; f__Chitinophagaceae;g__Chitinophaga	0.24	0.01	0.00	0.00	0.00	0.00	0.00	0.00	0.00	0.01
Bacteroidetes;c__Sphingobacteriia;o__Sphingobacteriales; f__Flexibacteraceae;g__Hymenobacter	1.00	0.03	0.01	0.00	0.01	0.00	0.00	0.00	0.00	0.00
Bacteroidetes;c__Sphingobacteriia;o__Sphingobacteriales; f__Sphingobacteriaceae;g__Pedobacter	0.32	0.03	0.00	0.00	0.00	0.00	0.00	0.00	0.00	0.00
Bacteroidetes;c__Sphingobacteriia;o__Sphingobacteriales; f__Sphingobacteriaceae;g__Sphingobacterium	0.77	0.02	0.00	0.00	0.00	0.00	0.01	0.00	0.00	0.00
Chloroflexi;c__Anaerolineae;o__Anaerolineales; f__Anaerolinaceae;g__SHD-231	0.01	0.10	0.27	0.28	0.21	0.13	0.24	0.27	0.34	0.30
Fibrobacteres;c__Fibrobacteria;o__Fibrobacterales; f__Fibrobacteraceae;g__Fibrobacter	0.07	0.32	1.32	2.07	0.62	2.28	6.79	7.51	6.19	2.01
Firmicutes;c__Bacilli;o__Bacillales; f__Bacillaceae;g__Bacillus	62.05	0.04	0.00	0.00	0.00	0.00	0.00	0.00	0.00	0.00
Firmicutes;c__Bacilli;o__Lactobacillales; f__Streptococcaceae;g__Streptococcus	0.00	0.00	0.03	0.01	0.03	0.21	0.00	0.01	0.00	0.00
Firmicutes;c__Clostridia;o__Clostridiales; f__Clostridiaceae;g__Clostridium	0.19	0.98	1.76	2.24	1.86	1.54	2.17	2.64	3.27	2.33
Firmicutes;c__Clostridia;o__Clostridiales; f__Dehalobacteriaceae;g__Dehalobacterium	0.00	0.03	0.11	0.40	0.07	0.07	0.14	0.15	0.33	0.58
Firmicutes;c__Clostridia;o__Clostridiales; f__Lachnospiraceae;g__Anaerostipes	0.02	0.18	0.49	0.43	0.30	0.33	0.31	0.25	0.25	0.40
Firmicutes;c__Clostridia;o__Clostridiales; f__Lachnospiraceae;g__Butyrivibrio	0.22	2.21	3.33	3.49	4.40	3.11	2.68	2.70	2.81	3.34
Firmicutes;c__Clostridia;o__Clostridiales; f__Lachnospiraceae;g__Coprococcus	0.05	0.46	0.98	0.99	1.02	0.91	0.76	0.72	0.75	0.88
Firmicutes;c__Clostridia;o__Clostridiales; f__Lachnospiraceae;g__Moryella	0.01	0.12	0.20	0.21	0.18	0.13	0.14	0.10	0.15	0.14
Firmicutes;c__Clostridia;o__Clostridiales; f__Lachnospiraceae;g__Pseudobutyrivibrio	0.01	0.49	0.83	0.70	0.66	1.45	0.65	0.76	0.61	0.73
Firmicutes;c__Clostridia;o__Clostridiales; f__Ruminococcaceae;g__Oscillospira	0.01	0.37	0.40	0.53	0.47	0.28	0.29	0.41	0.50	0.67
Firmicutes;c__Clostridia;o__Clostridiales; f__Ruminococcaceae;g__Ruminococcus	0.08	1.90	3.23	2.72	2.52	2.74	2.34	2.46	2.64	2.82
Firmicutes;c__Clostridia;o__Clostridiales; f__Veillonellaceae;g__Anaerovibrio	0.00	0.02	0.13	0.06	0.08	0.07	0.05	0.08	0.07	0.05
Firmicutes;c__Clostridia;o__Clostridiales; f__Veillonellaceae;g__Selenomonas	0.01	0.16	0.28	0.20	0.19	0.43	0.10	0.09	0.05	0.12
Firmicutes;c__Clostridia;o__Clostridiales; f__Veillonellaceae;g__Succiniclasticum	0.05	0.20	0.39	0.38	0.35	0.54	0.30	0.32	0.32	0.45
Firmicutes;c__Erysipelotrichi;o__Erysipelotrichales; f__Erysipelotrichaceae;g__L7A_E11	0.00	0.04	0.04	0.16	0.08	0.07	0.09	0.12	0.25	0.13
Proteobacteria;c__Alphaproteobacteria;o__Rhizobiales; f__Hyphomicrobiaceae;g__Devosia	1.62	0.08	0.03	0.01	0.06	0.02	0.02	0.01	0.02	0.00
Proteobacteria;c__Alphaproteobacteria;o__Rhizobiales; f__Methylobacteriaceae;g__Methylobacterium	0.32	20.14	0.00	0.02	0.00	0.02	0.00	0.00	0.00	0.01
Proteobacteria;c__Alphaproteobacteria;o__Rhizobiales; f__Rhizobiaceae;g__Agrobacterium	0.74	0.04	0.09	0.00	0.03	0.02	0.00	0.00	0.00	0.06
Proteobacteria;c__Alphaproteobacteria;o__Rhizobiales; f__Rhizobiaceae;g__Rhizobium	0.05	0.00	0.00	0.00	0.00	0.00	0.00	0.00	0.00	0.01
Proteobacteria;c__Alphaproteobacteria;o__Rhodobacterales; f__Rhodobacteraceae;g__Paracoccus	0.15	0.01	0.01	0.00	0.03	0.00	0.00	0.00	0.00	0.01
Proteobacteria;c__Alphaproteobacteria;o__Rhodobacterales; f__Rhodobacteraceae;g__Rhodobacter	0.18	0.01	0.00	0.00	0.00	0.00	0.00	0.00	0.00	0.00
Proteobacteria;c__Alphaproteobacteria;o__Rhodospirillales; f__Acetobacteraceae;g__Roseomonas	0.38	0.03	0.01	0.01	0.03	0.02	0.00	0.00	0.00	0.02
Proteobacteria;c__Alphaproteobacteria; o__Sphingomonadales;f__Sphingomonadaceae; g__Sphingomonas	0.98	0.08	0.01	0.03	0.06	0.01	0.00	0.01	0.00	0.01
Proteobacteria;c__Deltaproteobacteria;o__Desulfovibrionales; f__Desulfovibrionaceae;g__Desulfovibrio	0.00	0.04	0.24	0.14	0.13	0.20	0.20	0.26	0.18	0.17
Proteobacteria;c__Gammaproteobacteria;o__Aeromonadales; f__Succinivibrionaceae;g__Ruminobacter	0.04	0.58	0.83	0.77	0.50	0.69	0.98	0.48	0.53	0.59
Proteobacteria;c__Gammaproteobacteria; o__Enterobacteriales;f__Enterobacteriaceae; g__Erwinia	0.61	0.07	0.01	0.00	0.00	0.00	0.00	0.00	0.00	0.01
Proteobacteria;c__Gammaproteobacteria; o__Pseudomonadales;f__Pseudomonadaceae; g__Pseudomonas	2.46	0.12	0.03	0.02	0.09	0.01	0.00	0.00	0.00	0.02
Proteobacteria;c__Gammaproteobacteria; o__Xanthomonadales;f__Xanthomonadaceae; g__Stenotrophomonas	0.15	0.00	0.01	0.00	0.02	0.00	0.00	0.00	0.00	0.01
Spirochaetes;c__Spirochaetes;o__Sphaerochaetales; f__Sphaerochaetaceae;g__Sphaerochaeta	0.01	0.83	0.56	0.62	0.54	0.62	0.37	0.34	0.48	0.49
Spirochaetes;c__Spirochaetes;o__Spirochaetales; f__Spirochaetaceae;g__Treponema	0.22	0.83	1.11	0.81	0.52	1.81	3.89	4.06	2.92	1.42
Synergistetes;c__Synergistia;o__Synergistales; f__Dethiosulfovibrionaceae;g__Pyramidobacter	0.00	0.01	0.08	0.02	0.03	0.09	0.07	0.06	0.11	0.04
Tenericutes;c__Mollicutes;o__Anaeroplasmatales; f__Anaeroplasmataceae;g__Anaeroplasma	0.02	0.49	0.49	0.27	0.63	0.85	0.63	0.28	0.15	0.20
Tenericutes;c__Mollicutes;o__Anaeroplasmatales; f__Anaeroplasmataceae;g__RFN20	0.07	5.11	2.30	2.31	5.27	1.64	1.40	1.37	1.22	1.98
Bacteria genera_<0.1%	0.81	0.30	0.37	0.30	0.40	0.36	0.23	0.22	0.30	0.40
Unclassified bacteria genera	24.16	40.37	61.27	60.08	60.98	55.00	54.71	56.78	62.09	62.43
**ARCHAEA**										
Euryarchaeota;c__Methanobacteria;o__Methanobacteriales; f__Methanobacteriaceae;g__Methanobrevibacter	0.01	0.27	0.56	0.45	0.86	0.33	0.21	0.19	0.30	0.66
Archaeal genera <0.1%	0.00	0.03	0.11	0.06	0.09	0.10	0.02	0.02	0.01	0.07

**Table 8:** **Community composition of switchgrass-adherent microbiome at the phylum level based on 16S rRNA pyrotag data**.

	**Percentage of sequences**									
	**0 h**	**0.5 h**	**1 h**	**2 h**	**4 h**	**6 h**	**16 h**	**24 h**	**48 h**	**72 h**
**BACTERIA**										
Firmicutes	65.27	25.57	42.79	44.25	44.47	39.93	38.41	40.41	45.00	44.78
Proteobacteria	22.75	25.36	5.91	3.39	5.52	5.91	4.26	2.67	2.41	2.96
Bacteroidetes	7.29	34.09	32.02	36.84	28.02	37.00	35.08	35.37	32.47	34.54
Actinobacteria	3.79	0.60	0.39	0.48	0.70	0.28	0.20	0.23	0.36	0.43
Cyanobacteria	0.01	0.90	1.48	0.80	1.30	0.82	0.97	0.65	0.33	0.95
Spirochaetes	0.23	1.80	2.07	1.66	1.24	2.72	4.42	4.61	3.75	2.21
Tenericutes	0.21	7.46	6.18	5.10	10.34	5.78	4.59	3.47	3.17	4.61
Fibrobacteres	0.14	0.36	1.35	2.14	0.63	2.30	6.86	7.54	6.23	2.11
Elusimicrobia	0.03	0.40	0.39	0.23	0.30	0.25	0.29	0.26	0.26	0.27
Verrucomicrobia	0.01	0.37	0.52	0.50	0.58	0.36	0.32	0.26	0.27	0.64
Lentisphaerae	0.02	0.34	0.79	0.55	0.58	0.52	0.59	0.39	0.58	0.72
Planctomycetes	0.01	0.25	0.54	0.51	0.50	0.26	0.29	0.39	0.62	0.60
Chloroflexi	0.01	0.13	0.36	0.34	0.23	0.16	0.27	0.29	0.39	0.36
TM7	0.02	0.06	0.17	0.03	0.26	0.05	0.05	0.00	0.03	0.03
Synergistetes	0.00	0.03	0.18	0.06	0.06	0.13	0.12	0.09	0.15	0.10
Bacteria phyla <0.1%	0.04	0.04	0.07	0.04	0.04	0.01	0.05	0.05	0.11	0.10
Unclassified bacteria phyla	0.15	1.95	4.11	2.57	4.28	3.11	2.72	3.11	3.56	3.86
**ARCHAEA**										
Euryarchaeota	0.01	0.29	0.67	0.50	0.96	0.43	0.23	0.22	0.31	0.73

**Figure 3:**
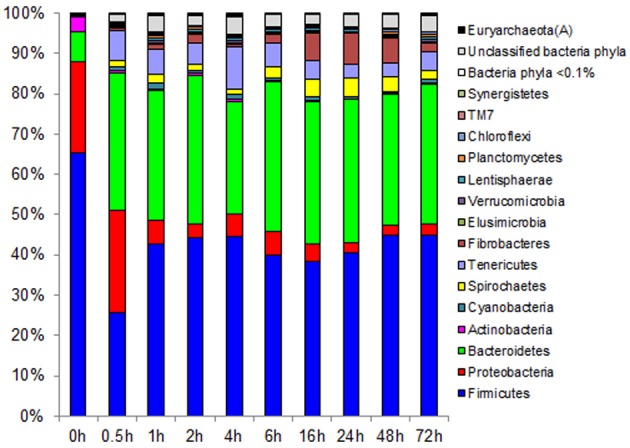
**Effect of rumen incubation on relative abundance of bacterial and archaeal phyla of switchgrass-adherent microbiome**. Relative OTU abundance of rarefied 16S rRNA gene 454-pyrotag data. Taxonomy was assigned based on Greengenes (McDonald et al., 2012).

Observed decreases in *Firmicutes* were driven by a 460-fold reduction in the *Bacilli* (mostly *Bacillus* spp.), which initially accounted for 97% of the *Firmicutes* phylum and 63% of all bacteria prior to rumen incubation. Decreases in *Bacilli* were partially offset by large increases in the *Clostridia* including the *Lachnospiraceae* (particularly *Butyrivibrio* spp., *Pseudobutyrivibrio* spp., and *Clostridium* spp.) and the *Ruminococcaceae* (almost entirely *Ruminococcus* spp.) families. The *Clostridia* continued to increase to become the predominant class and made *Firmicutes* the predominant phylum after a brief peak in *Proteobacteria* abundance within the initial 30 min of rumen-incubation (Figure 3, Tables 6, 8). The switchgrass-associated microbiota stabilized at the phylum level after 1 h of rumen incubation (Figure 3), and only modest phylum- and class-level changes were observed for most taxonomic groups between 1 and 72 h of rumen incubation (Tables 6, 8). The only exceptions to this observation were the *Fibrobacteres* and *Spirochaetes*, which both increased considerably in abundance in samples collected between 6 and 16 h, peaking around 24 h before returning to their 1 h levels after a total of 72 h of rumen-incubation (Figure 3, Table 8). Each of these phylum-level changes was driven by a single genus: the *Fibrobacteres* driven exclusively by *Fibrobacter*, while changes in the *Spirochaetes* were driven by *Treponema* (Table 7).

### Biomass-degradation coordinated with microbial colonization

**Figure 4:**
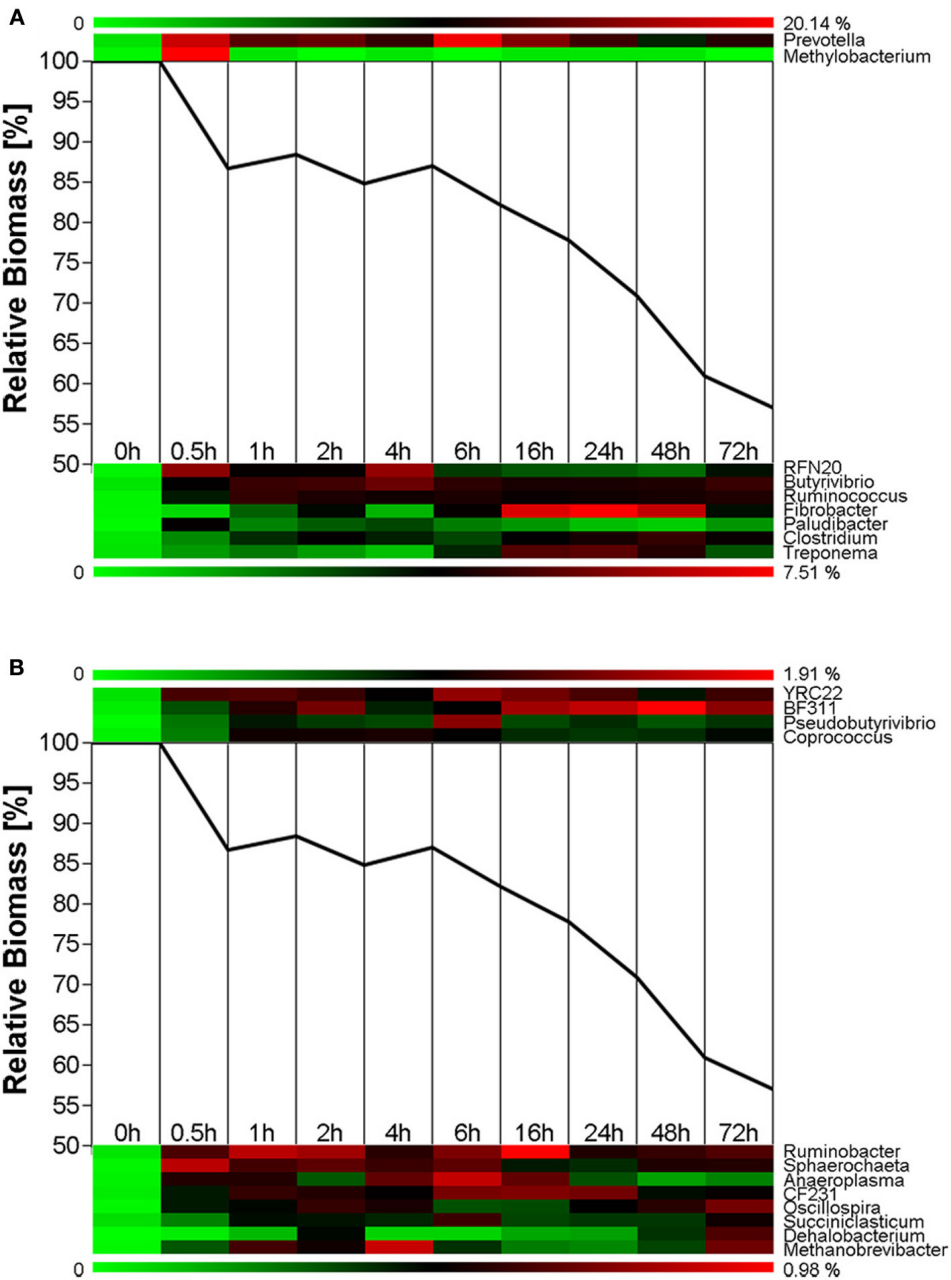
**Microbial succession and biomass-degradation during rumen-incubation**. Heat map show succession of genera recruiting >0.5% of the generated sequences. Line graphs show the relative dry mater change during rumen-incubation. **(A)** Upper panel shows genera with >10% relative abundance, lower panel shows genera with a relative abundance between 2 and 10%; **(B)** Upper panel shows genera with relative abundance between 1 and 2%, lower panel shows genera with a relative abundance between 0.5 and 1%.

**Figure 5:**
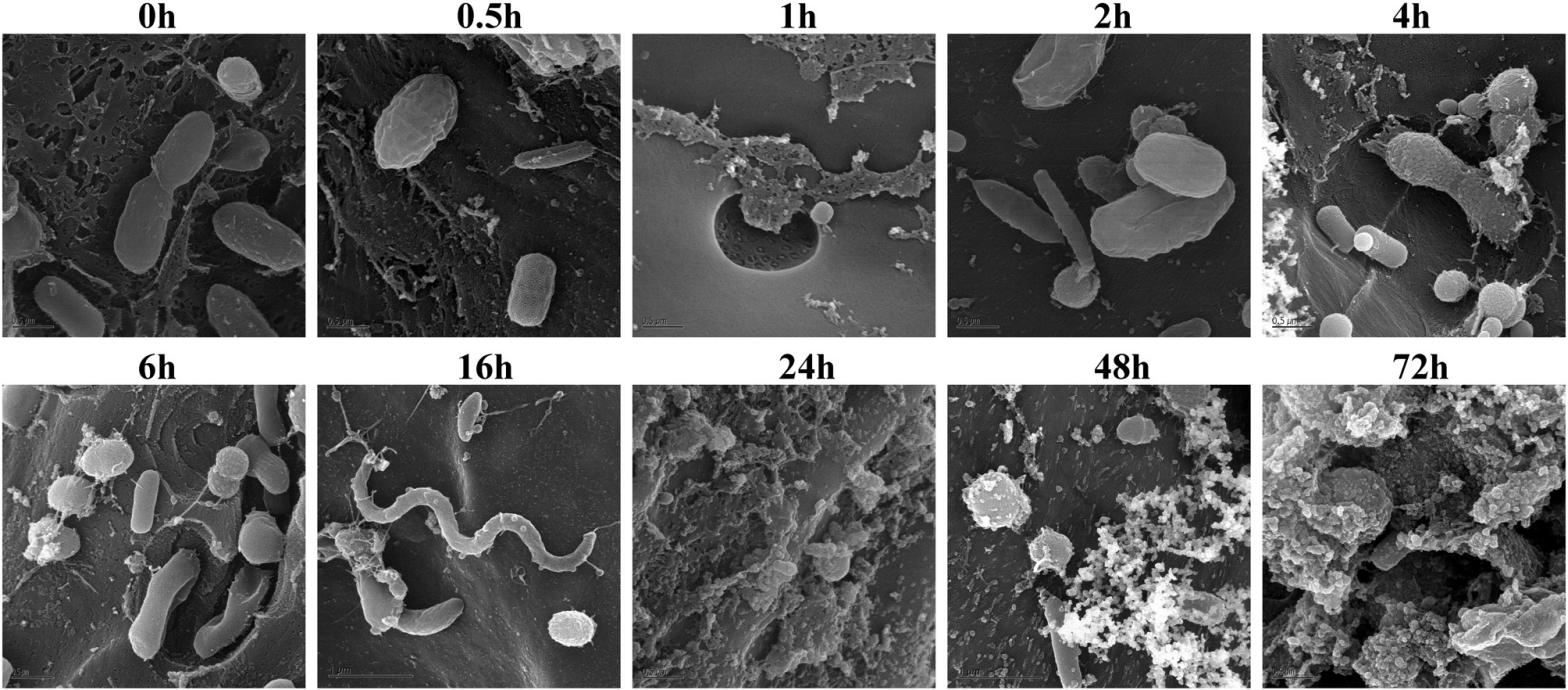
**Scanning Electron Microscopy of air-dried and rumen-incubated switchgrass samples and adherent microorganisms**. Scale bars indicate 0.5 μm.

Biomass degradation following rumen incubation appeared to be biphasic; each phase corresponding to notable changes in microbial communities. Biomass degradation began rapidly following rumen incubation with 13% of the total biomass being degraded within 30 min (Figure 4). Biomass-degradation then appeared to stall between 30 min and 4 h, with no significant change to biomass during this period. Corresponding to this period, we observed noticeable increases in *Methanobrevibacter* spp. and changes in some bacterial taxa. The *Butyrivibrio* spp. increased, while *Prevotella* spp. decreased between 30 min and 4 h. Following 4 h, biomass degradation proceeded somewhat linearly at a rate of ~0.81% biomass h^−1^ for the next 20 h of incubation, before gradually slowing over the following two measurements (~0.41% biomass h^−1^ between 24 and 48 h and 0.17% biomass h^−1^ between 48 and 72 h). The increase in rate of biomass degradation between 4 and 24 h occurred simultaneously with increased numbers of reads assigned to *Fibrobacter* and *Treponema* species (Figure 4, Table 7). Biomass-degradation and colonization of switchgrass was also detectable by SEM with significant fiber degradation being visible for samples incubated within the rumen for 24 h and longer (Figure 5).

## Discussion

In the present study we observed temporal shifts in the plant biomass-associated microbiota and in the rates of biomass degradation during the *in situ* rumen-incubation of dried switchgrass. Changes in the microbiome were in particular prevailing immediately within the first 30 min and after 4 h of rumen incubation. These observations are consistent with analogous trials on rumen-incubated fresh perennial ryegrass, where denaturing gradient gel electrophoresis (DGGE)-based analyses identified discrete microbial profiles at 0–2 h and after 4 h onwards (Huws et al., 2013). Notable similarities and differences were observed in several findings between these two studies. Consistent with our findings, Huws and colleagues reported increases in richness and diversity following rumen incubation (Huws et al., 2013). The authors of the former study also found the major ruminal genus *Prevotella* (Stevenson and Weimer, 2007) increased after 4 h. In our study, *Prevotella* spp. growth was biphasic, increasing dramatically within the first 30 min following rumen incubation and decreasing between 30 min and 4 h, before increasing again. In our study, *Prevotella* spp. were most abundant at 6 h and did not return to 4 h levels until after 24 h of incubation. In contrast to our observations, Huws et al. reported a loss of a band corresponding to *Treponema* spp. after 4 h rumen incubation (Huws et al., 2013), whereas we saw great increases in this genus between 6 and 16 h. This discrepancy likely reflects differences in analytical techniques (DGGE vs. next-generation sequencing), where loss of a single *Treponema* band may not reflect all members of that taxonomic group. Direct sequencing of 16S rRNA amplicons also facilitated the detection of a significant increase of *Methylobacterium* in switchgrass samples retrieved after 30 min of rumen incubation (Figure 4). Members of the genus *Methylobacterium* are strictly aerobic, can utilize C_1_ compounds such as formate, methanol and methaylamine and a variety of C_2_ (including acetate), C_3_, and C_4_ compounds (Green, 2006) as sole carbon source, and have been found in association with a variety of plants (Corpe, 1985). We hypothesize that members of the *Methylobacterium* were associated with the switchgrass fibers and introduced into the rumen, where they metabolized common fermentation intermediates, such as formate and acetate (Sabine and Johnson, 1964; Hungate et al., 1970), until all oxygen that might have been introduced with the ground switchgrass was depleted. Overall many similarities exist between the two studies and may indicate that the temporal changes in microbiota during the first 30 min following rumen-incubation and then after 4 h may be common features of rumen-microbial digestion among multiple plant species in various states (fresh vs. dried). Changes in the microbiota corresponded with differences in the rates of switchgrass degradation. The rate of switchgrass degradation was greatest within the first 30 min of rumen-incubation during which 13% of the total biomass was lost. This may reflect rapid utilization of soluble sugars and other easily fermentable nutrients that were unabated by proximal H_2_ partial pressures. However, the rate of degradation stalled between 30 min and 4 h. During this period we observed a dramatic increase in methanogen density such that the total abundance of methanogens at 4 h post-incubation increased 3.3-fold compared to 30 min incubation. We hypothesize that these two observations were linked and related to a dramatic increase in proximal H_2_ partial pressure caused by the rapid initial fermentation of easily accessible plant polysaccharides. Methanogen numbers swelled, most likely in response to the hypothesized high H_2_ partial pressures and were eventually able to overcome these and create an environment that was favorable to the energetically-efficient growth of the fibrolytic microbial consortia, as has been proposed based on *in vitro* evidence (Stams, 1994; Ishii et al., 2006). Although the community was largely established by 1 h, as evidenced by less dramatic changes in the overall microbiota 16S rRNA profile following this period, it was not until after 4 h before their collective metabolism was operating efficiently.

In summary the findings presented here support the hypothesis that the hydrogenotrophic metabolism of methanogens is an essential part of the fibrolytic activity of the rumen ecosystem and is essential for the degradation of the more recalcitrant plant polysaccharides by rumen microbes. Additional *in situ* work during which methane emission, fiber composition and community are monitored more stringently will be essential for obtaining a better holistic understanding of the rumen microbiome and the contribution of particular community members to the fibrolytic and methanogenic rumen phenotype.

## Author contributions

Conceived and designed the experiments: Matthias Hess, Susannah G. Tringe, and Roderick I. Mackie. Performed the experiments: Matthias Hess, Hailan Piao, Roderick I. Mackie. Generated and analyzed the data: Matthias Hess, Hailan Piao, Medora Lachman, Stephanie Malfatti, Alexander Sczyrba, Bernhard Knierim, Manfred Auer, Susannah G. Tringe, and Carl J. Yeoman. Wrote the paper: Matthias Hess, Hailan Piao, Medora Lachman, and Carl J. Yeoman.

### Conflict of interest statement

The authors declare that the research was conducted in the absence of any commercial or financial relationships that could be construed as a potential conflict of interest.
